# TMKit: a Python interface for computational analysis of transmembrane proteins

**DOI:** 10.1093/bib/bbad288

**Published:** 2023-08-17

**Authors:** Jianfeng Sun, Arulsamy Kulandaisamy, Jinlong Ru, M Michael Gromiha, Adam P Cribbs

**Affiliations:** Nuffield Department of Orthopedics, Rheumatology, and Musculoskeletal Sciences, Botnar Research Centre, University of Oxford, Headington, Oxford OX3 7LD, UK; Department of Biotechnology, Bhupat and Jyoti Mehta School of BioSciences, Indian Institute of Technology Madras, Chennai 600036, Tamil Nadu, India; Chair of Prevention of Microbial Diseases, School of Life Sciences Weihenstephan, Technical University of Munich, 85354 Freising, Germany; Department of Biotechnology, Bhupat and Jyoti Mehta School of BioSciences, Indian Institute of Technology Madras, Chennai 600036, Tamil Nadu, India; Nuffield Department of Orthopedics, Rheumatology, and Musculoskeletal Sciences, Botnar Research Centre, University of Oxford, Headington, Oxford OX3 7LD, UK

**Keywords:** bioinformatics, transmembrane proteins, sequence analysis, structural biology, protein interaction interfaces, feature extraction

## Abstract

Transmembrane proteins are receptors, enzymes, transporters and ion channels that are instrumental in regulating a variety of cellular activities, such as signal transduction and cell communication. Despite tremendous progress in computational capacities to support protein research, there is still a significant gap in the availability of specialized computational analysis toolkits for transmembrane protein research. Here, we introduce TMKit, an open-source Python programming interface that is modular, scalable and specifically designed for processing transmembrane protein data. TMKit is a one-stop computational analysis tool for transmembrane proteins, enabling users to perform database wrangling, engineer features at the mutational, domain and topological levels, and visualize protein–protein interaction interfaces. In addition, TMKit includes seqNetRR, a high-performance computing library that allows customized construction of a large number of residue connections. This library is particularly well suited for assigning correlation matrix-based features at a fast speed. TMKit should serve as a useful tool for researchers in assisting the study of transmembrane protein sequences and structures. TMKit is publicly available through https://github.com/2003100127/tmkit and https://tmkit-guide.herokuapp.com/doc/overview.

## INTRODUCTION

Transmembrane proteins comprise approximately 20–30% of the human genome [1] and are therapeutic targets of more than 50% commercially released drugs [2]. Despite the biological significance, functional studies of transmembrane proteins have been hampered by an acute shortage of their experimentally determined structures that make up only 2–3% of all of the structures deposited in the protein data bank (PDB) database [3, 4]. This scarcity of structural data underscores the need for computational approaches to assist in the analysis and understanding of functions of these critical biomolecules. A handful of databases specific to transmembrane proteins have been generated to make high-quality 3D structures available for computational analysis [5–8]. The protein data bank of transmembrane proteins (PDBTM) stands out as a trustworthy data source that comprehensively collects membrane plane-placed and error-corrected transmembrane protein assemblies [6, 9], each being subject to the determination of nine possible topologies using a structure-based algorithm TMDET [10]. Although PDBTM contains rich structural and topological information, there is currently no publicly available computational tool for large-scale topological annotation, file retrieval and format conversion. This limitation hinders the widespread use of PDBTM in the research community.

Most existing computational tools focus only on addressing object-specific issues, such as database handling [11–13], feature generation [14, 15], and visualization [16, 17]. There is a shortage of open-source software for one-stop computational analysis, which covers the modules above. In addition, it is known that the oligomerization of transmembrane proteins for maintaining and regulating many cellular activities, such as signal transduction and immune responses, necessitates a large number of protein–protein interactions (PPIs) occurring on cell membranes [18]. However, there is no publicly available tool for visualizing PPI interfaces between them to better support the biological interpretation. In summary, the computational community has been, while filled with toolkits for protein science [19–22], devoid of those specialized for transmembrane proteins.

Here, we present TMKit, a scalable Python toolkit that comprises various function modules for the analysis of transmembrane protein sequences, structures and functions. While many function modules in TMKit are specialized for transmembrane protein-related problems, they can also be applied to other kinds of proteins. One of the standout features of TMKit is its visualization module, which facilitates the biological validation, interpretation and translation of known and predicted PPI interfaces. In addition, TMKit also incorporates seqNetRR, a high-performance computing library that simplifies the construction of residue–residue connections and speeds up feature assignment, enabling faster feature analysis and machine learning modeling. Overall, TMKit is a helpful toolkit for computational studies of transmembrane proteins.

## MATERIALS AND METHODS

### Functionality of TMKit

**Figure 1:**
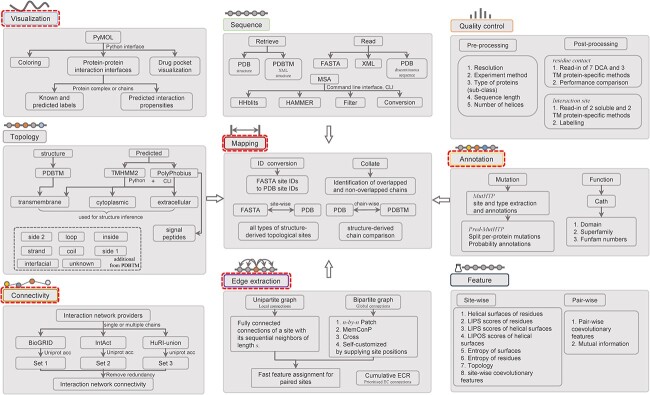
Functionality of TMKit. *Visualization*, *Mapping*, *Annotation*, *Connectivity*, and *Edge extraction*modules in TMKit are considered unique compared to existing tools.

TMKit provides nine function modules to handle several transmembrane protein sequence and structural analysis problems, including *visualization*, *sequence, quality control*, *topology*, *mapping*, *annotation*, *connectivity*, *edge extraction* and *feature* (Figure 1).

#### Visualization

**Figure 2:**
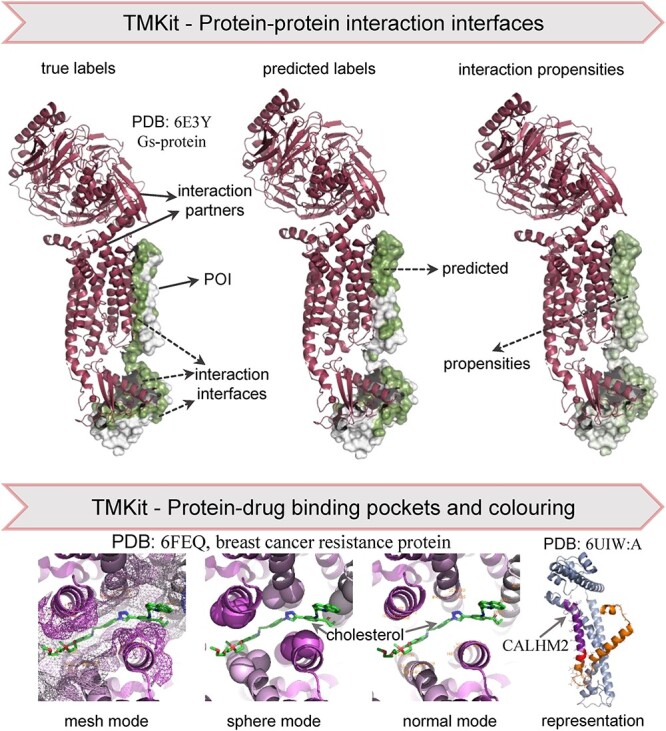
Visualization of transmembrane PPI interfaces, protein–drug binding pockets and coloring representations in TMKit.

Identification of PPI interfaces is critical to understand the biological processes governed by proteins. Direct visualization of the PPI interfaces on 3D structures can facilitate their localization at the atomic coordinate level. However, until now, there has been a lack of publicly available programs that allow the one-stop visualization of structural details of PPIs. TMKit is an open-source toolkit that enables access to the PPI interfaces by taking as input the structure of a protein of interest (POI) and a list of probabilities of residue sites to be involved in interactions. The program can automatically generate the label- or propensity-based PPI interfaces in between a POI and its interacting proteins (or its larger complex), which can be visualized in PyMOL [23]. For instance, we show in Figure 2 the PPI interfaces of chain E of the human calcitonin gene-related peptide (CGRP) with interacting chains P and R [24]. Given the pharmaceutical importance of membrane proteins, we additionally provide a one-stop service for visualization of close-ups of protein–drug binding pockets in a few ways, such as using the mesh or sphere to show the binding landscapes.

#### Sequence

The sequence pre-processing module is a fundamental component of TMKit, designed to handle sequence reading in diverse formats, sequence retrieval from various sources and multiple sequence alignment (MSA) generation. It can retrieve sequences from PDB and PDBTM, parse sequences from FASTA, structure and XML files, and stitch discontinuous residues together from PDB structures (see [25] for residues in unstructured regions). TMKit contains Python wrappers of the HHblits [26] and HAMMER [27] commands for further generating MSAs of a protein sequence, which can then be filtered according to a certain identity threshold between homologs or converted to another version (e.g. Stockholm format). TMKit allows the extraction of single residues or residue pairs from a protein sequence in a given sequence separation *k*. A sliding window of any size applied to each of the single residues or residue pairs can further generate a list of neighbor-surrounded residues or residue pairs in sequence context.

#### Quality control

The *quality control* module generates a set of protein sequences with sufficient quality, ensuring that they meet necessary criteria for conducting rigorous statistical analysis or prediction tasks. This module evaluates various conditions, including the experimentation methods used, resolution, subclass and sequence length, to qualify proteins in bulk. The uniqueness of this process is the incorporation of topology information derived from structures or predicted by programs, such as the number or the length of transmembrane α-helices. This information is critical for distinguishing between bitopic or other types of polytopic proteins, which is useful for downstream analysis.

#### Topology

Transmembrane protein topologies are vital to their structures and functions [28, 29]. For example, the α-helical-buddle embedded within membranes is highly conserved as a structural basis for the formation of a large membrane protein complex [30] and the extramembranous regions of G protein-coupled receptors (GPCRs) are functionally important as a checkpoint to interact with external molecules [31]. TMKit incorporates a comprehensive set of algorithms dedicated to determining the protein topology. As of now, the 3D atomic coordinates of membrane proteins in the RCSB PDB are primarily obtained from crystallized structures in the absence of membrane lipid bilayers. The localization of the membrane lipid bilayers can be deduced by the TMDET algorithm and used to indicate the positions of transmembrane segments spanning transmembrane regions [32]. The positions are stored in XML format through the TMDET (http://tmdet.enzim.hu/) [10] or PDBTM server (http://pdbtm.enzim.hu/) [6]. However, the positions do not reveal structurally deduced cytoplasmic or extracellular regions. To make this information available, TMKit implements a method, which was first proposed in the MBPred work [33] and applied later in the DeepTMInter work [34], to redistribute each TMDET’s non-TM topology with either cytoplasmic or extracellular label by stacking up against topologies predicted by an external method such as TMHMM. Also, TMKit can be used to obtain more detailed non-TM topologies, that is, side 1, side 2, strand, coil, inside, loop and interfacial. Besides the structure-derived topologies, TMKit also supplies predicted topologies by embedding TMHMM and PolyPhobius running on the command line interface (CLI) and within Python.

#### Mapping

Identifier mapping between structural and sequence data (e.g. PDB residue IDs and FASTA residue IDs) is an important technical premise to guarantee the correct interpretation of biological findings. Often, residues in FASTA format are imperfectly aligned with those from the PDB structure, as experimentally resolved residues in its crystal structure can become discontinuous due to various challenges associated with the crystallization process [25]. The mapping module plays a crucial role in scenarios where it is necessary to convert from PDB residue IDs of topologies deduced by TMDET to FASTA residue IDs for sequence analysis, or to convert sequence-based residue IDs of predicted topologies to PDB residue IDs.

In addition, protein structures are rendered as the asymmetric units (ASUs) in their PDB files, which represent the smallest but most fundamental units that can be reassembled to the quaternary structures (i.e. biological assembly in oligomer form) through symmetric operations such as rotations and translations based on BIOMT records [35, 36]. An example is the RCSB PDB structure of a transferase for archaeal membrane lipid biosynthesis (PDB code: 5GUF), which consists of two chains biologically but only one chain stored with its coordinates [37]. In addition to applying the operations for obtaining the oligomer structure of a protein from its crystalline state, the information about the BIOMT annotations can, to cap it all, be error-prone due to the fact that these can be either defined by researchers or predicted by software (e.g. the PSQ server) [38, 39]. The accession IDs to the non-ASUs are often not documented in PDB or UniProt, which causes a problem to perform analysis through the information wherever needed. In TMKit, we therefore offer the collation of PDB chains that are not the ASU since it is of importance to convert it back in many application scenarios, where, for example, the accurate UniProt accession code is required to trawl through its PPI networks from PPI databases. This module is implemented by comparing RCSB PDB structures and TMDET-generated structures, given that the TMDET algorithm allows both biological unit recovery and error correction during the course of the membrane plane determination. Furthermore, TMKit allows mapping from PDB IDs to UniProt accession codes.

#### Annotation

Amino acid residues of transmembrane proteins to be involved in mutations and function domains can be annotated through the MutHTP [40], Pred-MutHTP [41] and Cath [42] databases.

#### Connectivity

Studying connections of a protein to others in a PPI network is of crucial importance to understand its biological role. There are a few well-established databases such as BioGRID [43], IntAct [44] and HuRI [45], which are enriched for PPIs of soluble proteins as well as transmembrane proteins of note. For example, interactions that transmembrane proteins are involved in account for about 40% of the human interactome map [34]. However, managing various sources and formats is posing a difficulty to unravel the connectivity of proteins. To address this and make it easier to use, TMKit first mines all unique interaction partners of a given list of TM proteins from individual PPI databases and then obtains the union of them by removing redundancy.

#### Edge extraction

Quantification and characterization of connections between residues are thought to be key to deciphering and understanding the mechanisms behind a biological network. However, handling a large number of connections is often computationally intractable, primarily due to time complexity involved. Progress in residue contact prediction and interaction site identification has shown that the utilization of neighboring information of residues or residue pairs can result in an improvement in prediction accuracy. We provide a high-performance computing library for extracting connections between residues by constructing bipartite and unipartite graphs (where residue connections are treated as edges) and assigning features in linear time with respect to the number of residues used. We particularly detail this in section *seqNetRR for extracting residue–residue connections*.

#### Feature

A set of transmembrane protein-specific and general-purpose features is provided by TMKit in support of machine learning modeling. We first incorporate LIPS [46], a method for identifying transmembrane helical interfaces with lipids, to generate the lipophilicity and entropy scores of residues located in seven helical faces defined upon heptad repeats (Figure 1). By establishing a scoring function that combines information about residue lipophilicity and entropy, this program can compute the LIPOS score (i.e. lipophilicity) to evaluate the propensity of a face being exposed to lipids. The score has been used as features or criteria in *ab initio* structural modeling [47, 48] and interaction site identification [33]. Moreover, considering the core role of coevolutionary features in structural biology, we then place a module in TMKit to gain access to the fast assignment of coevolutionary features to pairwise or site-wise residues. Besides, transmembrane protein topologies can also be considered as features and included in TMKit.

### seqNetRR for extracting residue–residue connections

We additionally developed seqNetRR, a high-performance computing library for constructing residue connections in sequence and contact-map context and performing coevolutionary feature assignment in linear time. This module is aimed at facilitating feature engineering, scheme design and organization for machine learning modeling of interactions/contacts between residues. Below, we demonstrate the conceptualization, construction and implementation of seqNetRR.

#### Definition of local and global residue–residue connections

**Figure 3:**
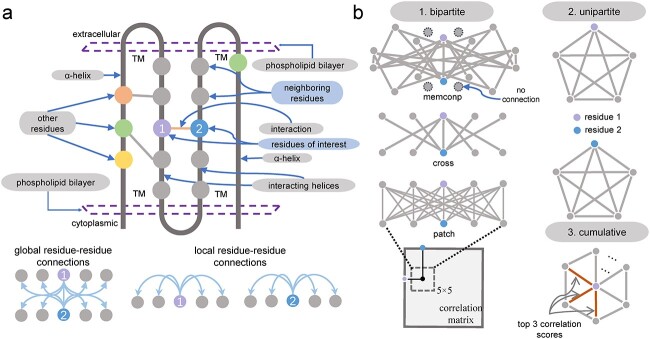
Schematic view of local and global residue–residue connections to be deemed as edges of graphs. (**A**) A transmembrane protein is shown in structure context within which a pair of helices are faced with each other and interact through interfacial residues. Residue 1 and residue 2 are two central residues, not adjacent in sequence but adjacent spatially. Other residues are their neighboring residues. (**B**) Different kinds of graphs with edges are taken as constraints to form connections in sequence (unipartite graphs) or contact-map (bipartite graphs) context. The cumulative graph shows the prioritized connections with the highest correlation scores between a residue of interest and others.

Connections between a residue of interest and its serially ordered neighboring residues are referred to as local residue–residue connections (LocRRCs for short). As seen in Figure 3A, in sequence context, LocRRCs are connections formed by residue 1 or 2 and other residues that serially flank it. Likewise, global residue–residue connections (GlobRRCs) are those formed by one residue of a residue pair of interest (including its neighbors, see section *Formulation of LocRRCs and GlobRRCs* for details) and the serially ordered neighboring residues of the other one residue of the residue pair (including itself, e.g. connections between residue 1 and the neighboring residues of residue 2). It is commonly seen in structural biology that coevolutionary features assigned to GlobRRCs are used to amplify the prediction performance of long-range residue contacts [49, 50]. Figure 3B shows that from a predicted contact map (i.e. statistically, a type of correlation matrix), residue connections in a square-shaped patch centering a pair of residues of interest are extracted first and then assigned features for a machine learning model to learn the characterization of the residue pair, which has been shown effective. The patch-like GlobRRCs reflect how strongly the two central residues connect their surrounding residues in contact-map context.

#### Formulation of LocRRCs and GlobRRCs

**Figure 4:**
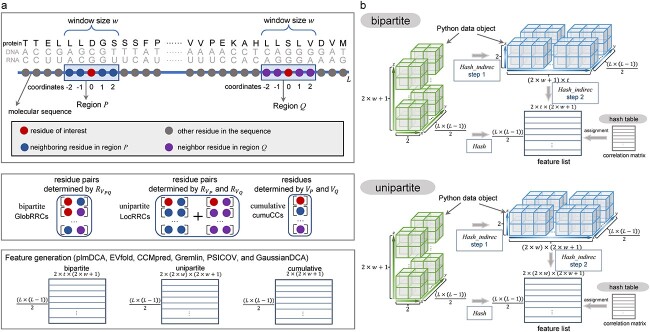
Illustration of construction of GlobRRCs/LocRRCs and feature assignment in seqNetRR of TMKit. (**A**) Extraction of window-placed connections between residues from a protein sequence of length $L$ (actually can be applied for DNA or RNA sequences). (**B**) Computational scheme of feature assignment. After applying a window of size $w$, a 3D Python data object is formed. The number of connections of residue pairs of interest is $\frac{\left(L\times \left(L-1\right)\right)}{2}$. Restricted by a bipartite graph with $t$ edges, the number of window-placed GlobRRCs is $\left(2\times w+1\right)\times t$. Restricted by a unipartite graph, the number of window-placed LocRRCs is $\left(2\times w\right)\times \left(2\times w+1\right)$. The feature assignment is performed by looking up residue positions per connection in a 2D hash table to take the corresponding values.

To describe connections between residues, we define a graph $G=\left(V,E\right)$ where $V$ is a set of vertices $\left\{{v}_1,{v}_2,\dots, {v}_L\right\}$ representing residues in a protein sequence of length $L$ and $E$ is a set of edges $\left\{{e}_1,{e}_2,\dots, {e}_m\right\}$ ($E\subset V\times V$) representing connections between residues. Residue ${v}_i$ has a neighbor ${v}_j$ if ${v}_j$ is a serially ordered residue surrounding ${v}_i$ in sequence context. As seen in Figure 4A, we place two equidistant sliding windows with the size $w$ to a given residue pair of interest ${v}_c^P$ and ${v}_c^Q$, respectively, to form two regions $P$ and $Q$. Any residue ${v}_i^P$ ($i=1,2,\dots, w$) from region $P$ and any residue ${v}_i^Q$ ($i=1,2,\dots, w$) from region $Q$ are elements of residue sets ${V}_P\left({V}_P\subset V\right)$ and ${V}_Q\left({V}_Q\subset V\right)$, respectively. Residues in blue in region $P$ are denoted as neighboring residues of ${v}_c^P$ in red, so are those of ${v}_c^Q$ in red in region $Q$. A LocRRC is formed by any two elements from set ${V}_P$ or set ${V}_Q$ and a GlobRRC is formed by an element from set ${V}_P$ and an element from set ${V}_Q$. All LocRRCs in regions $P$ and $Q$ are binary relations ${R}_{V_P}$ and ${R}_{V_Q}$, respectively, given by the *Cartesian products*  ${V}_P\times{V}_P$ and ${V}_Q\times{V}_Q$ as follows:


$${R}_{V_P}=\left\{<{v}_i^P,{v}_j^P>|{v}_i^P,{v}_j^P\in{V}_P\wedge{C}_{\mathrm{uni}}\right\}$$

$${R}_{V_Q}=\left\{<{v}_i^Q,{v}_j^Q>|{v}_i^Q,{v}_j^Q\in{V}_Q\wedge{C}_{\mathrm{uni}}\right\}$$

where $i,j=1,2,\dots, w$ and ${C}_{\mathrm{uni}}$ stands for a group of certain edges restricted by different kinds of unipartite graphs introduced below.

Similarly, all GlobRRCs are described as binary relations ${R}_{V_{PQ}}$, given by the *Cartesian products*  ${V}_P\times{V}_Q$, such that


$${R}_{V_{PQ}}=\left\{<{v}_i^P,{v}_j^Q>|{v}_i^P\in{V}_P\wedge{v}_j^Q\in{V}_Q\wedge{C}_{\mathrm{bi}}\right\}$$

where $i,j=1,2,\dots, w$ and ${C}_{\mathrm{bi}}$ stands for a group of certain edges restricted by different kinds of bipartite graphs introduced below.

#### Unipartite and bipartite graphs

For LocRRCs, a set of edges ${e}_1{e}_2\dots{e}_{t-1}{e}_t$ between a residue of interest and its neighboring residues is conceived as constraints to collectively characterize the sequence surroundings of this residue; for GlobRRCs, the set of edges is used to characterize the surroundings of a residue pair in contact-map context. Thus, the main role of making different bipartite and unipartite graphs is to provide informative edges to better characterize residues or residue pairs of interest. Several bipartite and unipartite graphs are introduced below as constraints ${C}_{uni}$ and ${C}_{bi}$ to form different kinds of LocRRCs and GlobRRCs.

##### Unipartite graphs

The two unipartite graphs $\left({V}_P+{V}_P,{E}_{\mathrm{uni}\_\mathrm{P}}\right)$ and $\left({V}_Q+{V}_Q,{E}_{\mathrm{uni}\_\mathrm{Q}}\right)$ for which edges between two vertices from the same set are only available [51] are used to demarcate LocRRCs. As shown in Figure 3B, we present an example of a unipartite graph with five vertices to be fully connected with each other. This is currently the unipartite graph scheme (i.e. ${C}_{\mathrm{uni}}$) to extract all LocRRCs for each central residue in seqNetRR of TMKit. Users can also impose other kinds of ${C}_{\mathrm{uni}}$ on the unipartite graph by specifying certain two coordinates as an edge between two residues, e.g. (0, 2) in region $P$ shown in Figure 4A.

##### Bipartite graphs

A bipartite graph $G$ is defined as $\left({V}_P+{V}_Q,{E}_{\mathrm{bi}}\right)$ [52], where there is no adjacency (i.e. connections) between nodes in either residue set ${V}_P$ or ${V}_Q$. We implemented three types of bipartite graphs, that is, *patch*, *memconp* and *cross* (Figure 3B). The *patch* graph consists of connections in a square patch centering a residue pair [50] and the *memconp* graph consists of possible connections between residues in a pair of helices arranged in a face-to-face manner, which were used in the DeepHelicon and MemConP studies [50, 53], rendering constraints ${C}_{bi}$ as ${v}_i^P\ne{v}_j^Q$ and $\left(i+a,j+b\right)\wedge \left(a,b\right)\in$ {(0,0), (0,1), (0,−1), (0,3), (0,−3), (0,4), (0,−4), (1,0), (−1,0), (3,0), (−3,0), (3,4), (−3,4), (3,−4), (−3,−4), (4,0), (−4,0), (4,3), (−4,3), (4,−3), (−4,−3), (4,4), (−4,4), (4,−4), (−4,−4)}, respectively. Another showcase is a *cross*-connected bipartite graph in which a node of interest in an edge set is connected to its counterparts in another edge set.

#### Prioritized connections with the highest correlation coefficients

In addition, seqNetRR implements a computing scheme of the cumulative correlation coefficient (cumuCC) for every single residue by summing the correlation coefficients of threshold-based co-evolutionarily important connections with other residues (Figure 3B). For example, in evolutionary biology, the sum of the $k$ highest evolutionary coupling scores of a residue can help evaluate the extent to which this residue bears evolutionary constraints (a type of correlation coefficient, also called evolutionary constraint ratio) [54]. This has been used as an important feature to gauge whether a residue is an interaction site in transmembrane proteins (see MBPred and DeepTMInter in [33, 34]). In a word, the cumuCC is given by


$$\mathrm{cumuCC}=\frac{1}{c}\times{\mathrm{CC}}_R$$

where ${\mathrm{CC}}_R$ stands for the sum of the $k$ highest correlation coefficients of residue connections involving the residue $R$ according to those ranked correlation coefficients in descending order and $c$ is the sum of the correlation matrix (e.g. generated by FreeContact [55]) over the number of residue pairs.

#### Implementation of feature assignment to LocRRCs and GlobRRCs

Efficiently assigning features to a substantial number of residue pairs is a crucial preliminary step before undertaking more sophisticated computational modeling. For a given protein sequence, a list of residues/residue pairs of interest are extracted first to be stored technically as the data object in green (placed with a window, if applicable, Figure 4B) and their LocRRCs or GlobRRCs are constructed then to be stored as the data object in blue. It may be more plausible in practice that a 3D Python data object that entirely stores LocRRCs or GlobRRCs is used finally for feature assignment, with the first dimension representing the number of LocRRCs/GlobRRCs per residue/residue pair of interest (*x*-axis), the second dimension representing the total number of residues/residue pairs of interest (*y*-axis) and the last dimension storing the sequence positions of every two residues (*z*-axis). However, this can bring additional computational cost because of the added operation of creating, storing and re-extracting LocRRCs or GlobRRCs. It worsens heavily when large proteins with exponentially increased residue pairs are used. The seqNetRR module of TMKit manages to assign features by directly using the data object in green to bypass the separate generation of the data object in blue. A hash table is built to look up the correlation coefficients (e.g. typically coevolutionary features) from a correlation matrix. We term this method *Hash*. Relatively, the feature assignment method that is involved in the separate generation is called *Hash_indirec.*

Looking up a correlation coefficient in the 2D hash table of a correlation matrix requires the time complexity of $O(1)+O(1)$ (i.e. row + column), namely, $O(1)$, where no loop operation (i.e. a single correlation coefficient) in the second dimension of the hash table negates further time consumption and meanwhile the load balancing problem [56] is ignored. The complexity of time required to look up a residue–residue connection in a window-free 3D data object of LocRRCs or GlobRRCs is $O(n)$, while it will grow to $O\left({n}^2\right)$ if a window is placed inside the 3D data object. The time complexity of feature assignment to LocRRCs or GlobRRCs rises to $O\left({n}^3\right)$ but is relevant with the choice of whether or not edge-enriched unipartite or bipartite graphs are used. It should be noticed that the time complexity does not exceed $O\left({n}^3\right)$ and should actually be ranging from $O(n)$ to $O\left({n}^3\right)$ because a small-size window and a graph with a few edges as constraints can vastly bring down the $O\left({n}^3\right)$ complexity.

**Figure 5:**
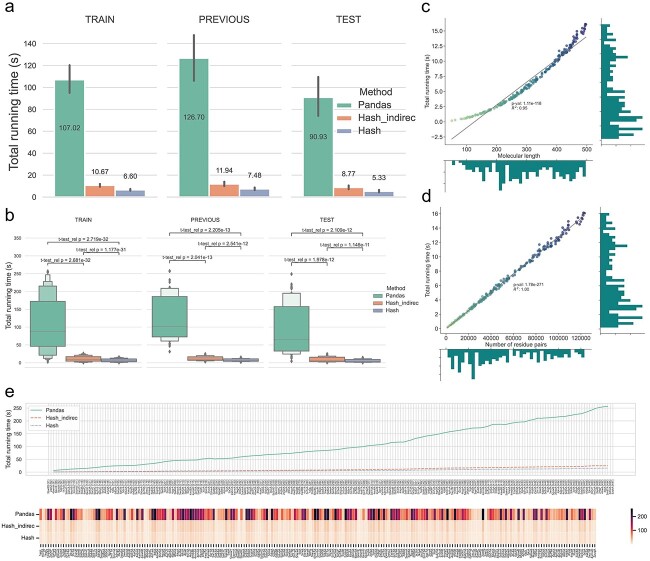
Computing performance of the construction of GlobRRCs and feature assignment. (**A**) Bar plot of the average running time of the two tasks. (**B**) Boxen plot of the total running time of the two tasks for each protein. (**C**) The total running time of the two tasks versus the molecular length per protein. (**D**) The total running time of the two tasks versus the number of residue pairs per protein. (**E**) Line plot and heatmap of each protein’s running time. *t-test_rel* for the paired *t*-test. To reduce noise, the running time has been grouped for data smoothing (a sliding window of size 5).

For comparison purposes, we additionally selected non-hashing look-up operations over a correlation matrix. These include the *pandas.at* method in Pandas [57] and the NumPy array indexing method [58]. In our case, the *pandas.at* method has been tested better than the *pandas*.*loc*, *pandas*.*ix* and *pandas*.*groupby* methods. Note that if the data object of LocRRCs or GlobRRCs is kept unchanged, introducing any further look-up operations over a correlation matrix can bring an additional cost of time complexity up to $O\left({n}^2\right)$ at worst.

## RESULTS

### Computing performance of feature assignment

We first evaluated the running time of different methods on assigning coevolutionary features to GlobRRCs restricted by the bipartite graph of a 5 × 5 patch (Figures 3B and5). The information about the benchmark datasets can be found in Supplementary Material, available online at http://bib.oxfordjournals.org/. We omitted the comparison with the NumPy array indexing method due to its extremely time-consuming nature. As shown in Figure 5A, the two methods involving hash tables outperform the Pandas *pandas.at* method in terms of the average running time, particularly with the *Hash* method taking 6.47 s (6.60 s for TRAIN, 7.48 s for PREVIOUS and 5.33 s for TEST). The paired *t*-tests confirm the statistically significant difference between the running time of every two methods (Figure 5B). The statistical test between *Hash_indirec* and *Hash* demonstrates that the time optimization relies on minimizing every individual unnecessary cost. Moreover, the run-time exhibits a strong correlation with the molecular length exponentially and the number of residue pairs linearly, as evidenced by significantly high R-squared values of 0.95 and 1, respectively. For instance, in the case of approximately 120 000 unrepeated residue pairs generated from a protein with a length of 500, our program completes the entire feature assignment in only 16 s.

**Figure 6:**
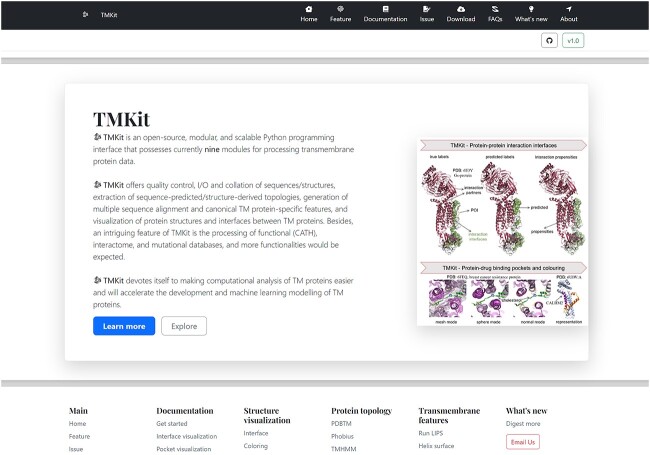
TMKit website interface.

Next, we examined the feature assignment to LocRRCs restricted by a 5-node unipartite graph (Figure 3B and Figure S1, available online at http://bib.oxfordjournals.org/). We included the comparison with the NumPy method because of its acceptable CPU time in handling LocRRCs. Similarly, the issue on the feature assignment to LocRRCs has also turned out to be trivial by using the *Hash* method, where the running time is only 1.63 s on average (1.64 s for TRAIN, 1.94 s for PREVIOUS and 1.30 s for TEST). The *Hash* method is 33-fold and 11-fold faster than the NumPy array indexing method and the Pandas pandas.at method, respectively.

We finally assessed the computing performance of assigning cumuCCs to individual residues using top-ranked residue pairs of protein lengths *L*/5, *L*/5, *L*/2, *L*, 1.5*L*, 2*L*, 5*L* and 10*L*, and found that our program performed the assignment of cumuCCs within 0.7 s for all the given thresholds (Figures S2–S4, available online at http://bib.oxfordjournals.org/).

### TMKit usage

TMKit is designed in an open-source, modular and scalable manner with 13 Python modules (9 function modules as displayed in section *Methods*) to handle various types of analyses of transmembrane protein sequences and structures. The TMKit functions can be accessed via the following modules: *tmkit.cath*, *tmkit.collate*, *tmkit.edge* (i.e. seqNetRR), *tmkit.feature*, *tmkit.mapping*, *tmkit.msa*, *tmkit.mut*, *tmkit.ppi*, *tmkit.qc*, *tmkit.rrc*, *tmkit.seq*, *tmkit.topo* and *tmkit.vs*. They are mainly used inline in Python. Note that in order to facilitate the development of TMKit, many functions provided by some of these modules work based on a few external Python packages or libraries, including LIPS [46], BioPandas [59], Scikit-learn [60] and Biopython [61].

### TMKit website

The TMKit website (https://tmkit-guide.herokuapp.com) provides the specification of the 13 Python modules where case studies are presented (Figure 6). Each case-study vignette shows the definition of input parameters and files, the running pipeline and the output.

## DISCUSSION

In this work, we present TMKit, a computational toolkit that offers numerous function modules for analyzing transmembrane protein sequences and structures. Many of its modules facilitate machine learning modeling of transmembrane proteins. TMKit’s visualization capabilities allow atomic-level visualization of known and predicted protein–protein interaction interfaces for rapid biological interpretation. In addition, TMKit integrates a high-performance computing library seqNetRR, which can construct various sets of residue connections and assign features in linear time with respect to the number of residue pairs used. The seqNetRR module is designed to permit fast computation for learning the surrounding information of residues/residue pairs of interest in machine learning modeling problems.

TMKit distinguishes itself from existing tools mainly in terms of its easy-to-use functions, fast running speed and relatively comprehensive coverage of a wide range of capabilities that handle transmembrane protein data in different formats. Transmembrane protein structures are routinely collected into a few bespoke databases, such as PDBTM [6]. Given the importance of these data sources and the widespread use of Python, TMKit is developed to access them swiftly and easily. The types of topologies in the lipid-anchored transmembrane proteins partly define their roles in biological processes. TMKit is imparted with a strong ability to process structure-derived or predicted transmembrane topological data. We realize that an increasing number of downstream protein analysis tasks (e.g. PPI prediction) require complicated procedures that can actually be taken apart into a batch of subtasks. In this aspect, users can flexibly engineer and layer the TMKit functions into the procedures of their analysis because TMKit is assembled modular, which paves the way for more advanced downstream analysis. Besides, existing tools in protein science are mostly subject specific [12, 17] and many of them are finally available as computational models. However, users may need to build their analysis frameworks or methods from scratch. In particular, to our knowledge, TMKit is the first open-source tool to visualize the structural details of PPI interfaces, which helps biological interpretation. Considering that TMKit provides an overarching solution for data munging at the sequence, domain, structural and PPI levels, it can also be used for analyzing other types of proteins.

The relentless progress in computational techniques has transformed the landscape of the entire structural biology field [62]. For example, AlphaFold2 [63] has predicted structures of an extensive repertoire of proteins for which their experimentally resolved structures are not available. The TMKit toolkit provides a function to access the AlphaFold2-predicted TM protein structures. In order to gain an understanding of biological processes that a predicted structure may be involved in, we further provide users with structural alignment based on the Foldseek webserver [64], which searches against its built-in structural databases. A promising future direction for TMKit to further enhance its functionalities is the extraction of structural features from the predicted structures.

**Key Points**

- TMKit is the first Python interface for providing a one-stop computational analysis of transmembrane protein sequences and structures.
- TMKit offers a range of flexible functions for visualizing atomic-level interaction/mutation interfaces between transmembrane proteins.
- TMKit includes a direct module to access many transmembrane protein-specific databases, e.g. PDBTM, for fast database wrangling.
- TMKit is well suited for extracting biological features of single/paired residues from large transmembrane proteins at a fast speed.
- TMKit allows for detailed topological, mutational and interaction annotations of transmembrane protein sites.

## Supplementary Material

Supplementary_File_bbad288

## Data Availability

The TMKit toolkit is publicly available at https://github.com/2003100127/tmkit and the documentation is available at https://tmkit-guide.herokuapp.com/doc/overview.

## Notes

**Jianfeng Sun** is a postdoctoral researcher at the University of Oxford, Oxford, UK. He has developed 2 computational methods for studying interactions between transmembrane protein sites. His main research interests are sequence analysis of transmembrane proteins, structural biology, computational biology, and nanopore and single-cell sequencing.

**Arulsamy Kulandaisamy** is a postdoctoral researcher in the Protein Bioinformatics Lab at the Department of Biotechnology of the IIT Bhupat and Jyoti Mehta School of BioSciences in Madras, India. He has developed more than 10 published databases or computational tools for transmembrane protein research. His main research interests are the prediction of mutations and stabilities of transmembrane protein sites with machine learning.

**Jinlong Ru** is a PhD candidate at the Technical University of Munich, Munich, Germany. His research interests are systems biology and computational metagenomics.

**M. Michael Gromiha** is a Professor at the Indian Institute of Technology Madras, India. His main research interests are structural analysis, prediction, folding and stability of globular and membrane proteins, protein interactions and development of bioinformatics databases and tools. He has published >250 research articles, 50 reviews, 7 editorials and 2 books entitled ‘*Protein Bioinformatics: From Sequence to Function*’ by Elsevier/Academic Press and ‘*Protein Interactions: Computational Methods, Analysis and Prediction*’ by World Scientific.

**Adam P. Cribbs** is an Associate Professor at the University of Oxford, Oxford, UK. His research is directed toward understanding disease biology from a functional genomics standpoint by developing computational approaches to advance our understanding of the mechanisms that regulate genes, proteins and biological pathways. His main research interests are systems biology, computational biology, nanopore and single-cell sequencing.
