# Supplementary material for: TMKit: a Python interface for computational analysis of transmembrane proteins
Source: Brief Bioinform. 2023 Aug 17;24(5):bbad288. doi: 10.1093/bib/bbad288 (PMC10516361; doi:10.1093/bib/bbad288)
Supplement: Supplementary_File_bbad288 [file supplementary_file_bbad288.docx]

***Supplementary materials***

**TMKit: a Python interface for computational analysis of transmembrane proteins**

Jianfeng Sun^1^, Arulsamy Kulandaisamy^2^, Jinlong Ru^3^, M. Michael Gromiha^2,#^, Adam P. Cribbs^1,#^

^1^ Botnar Research Centre, Nuffield Department of Orthopedics, Rheumatology, and Musculoskeletal Sciences, University of Oxford, Headington, Oxford OX3 7LD, UK

^2^ Department of Biotechnology, Bhupat and Jyoti Mehta School of BioSciences, Indian Institute of Technology Madras, Chennai 600036, Tamilnadu, India

^3^ Chair of Prevention of Microbial Diseases, School of Life Sciences Weihenstephan, Technical University of Munich, 85354 Freising, Germany

^#^To whom correspondence should be addressed. [adam.cribbs@ndorms.ox.ac.uk](mailto:adam.cribbs@ndorms.ox.ac.uk); [gromiha@iitm.ac.in](mailto:gromiha@iitm.ac.in)

**Benchmark datasets**

TRAIN, PREVIOUS, and TEST from Deephelicon [1] are used as benchmark datasets to gauge the running time of seqNetRR when it operates to assign coevolutionary features to GlobRRCs and LocRRCs. To manage seqNetRR to run in a reasonable time range, proteins with more than 500 residues are excluded, leaving 129, 37, and 53 proteins in the final three datasets, respectively. For each protein, the coevolutionary features are generated using the EVfold method [2] and obtained from <https://data.mendeley.com/datasets/k8tfvgftv3/2>.

**Statistical tests**

As the running time distributions of *Hash_indirec* and *Hash* are resembled due to the closed mean values, the normal independent-samples t-Test will not be susceptible to the difference of the two distributions. Therefore, the paired t-Test [3] is used to test the statistical difference.


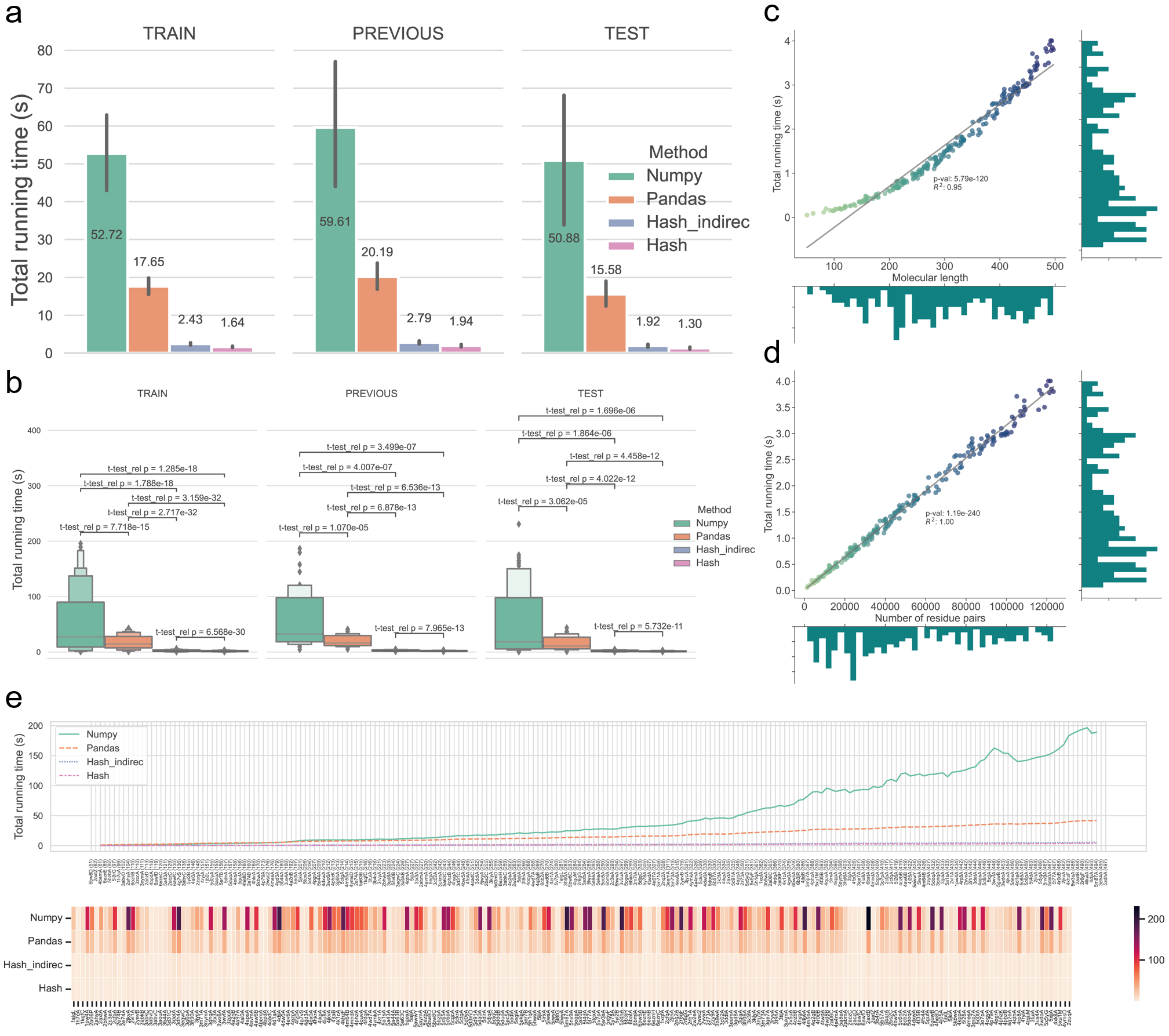


**Figure S1**. Computing performance of construction of LocRRCs and feature assignment. a. Bar plot of the average running time of the two tasks. b. Boxen plot of the total running time of the two tasks for each protein. c. The total running time of the two tasks *vs.* the molecular length per protein. d. The total running time of the two tasks *vs*. the number of residue pairs per protein. e. Line plot and heatmap of each protein’s running time. *t-test_rel* for the paired t-test. To reduce noise, the running time has been grouped for data smoothing (a sliding window of size 5).


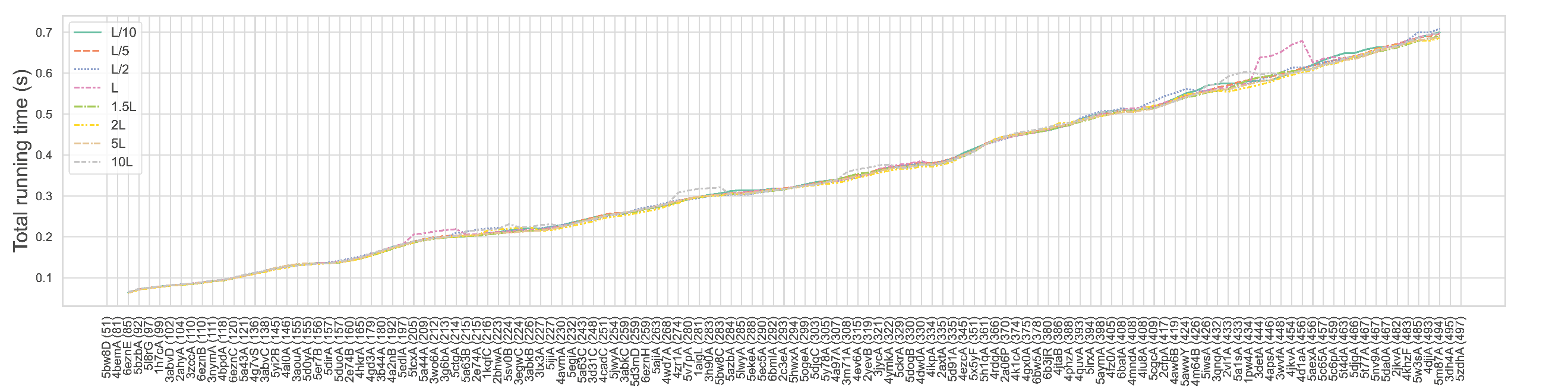


**Figure S2**. Total running time of generating cumuCCs per protein (TRAIN) at different cut-offs based on protein length *L*.


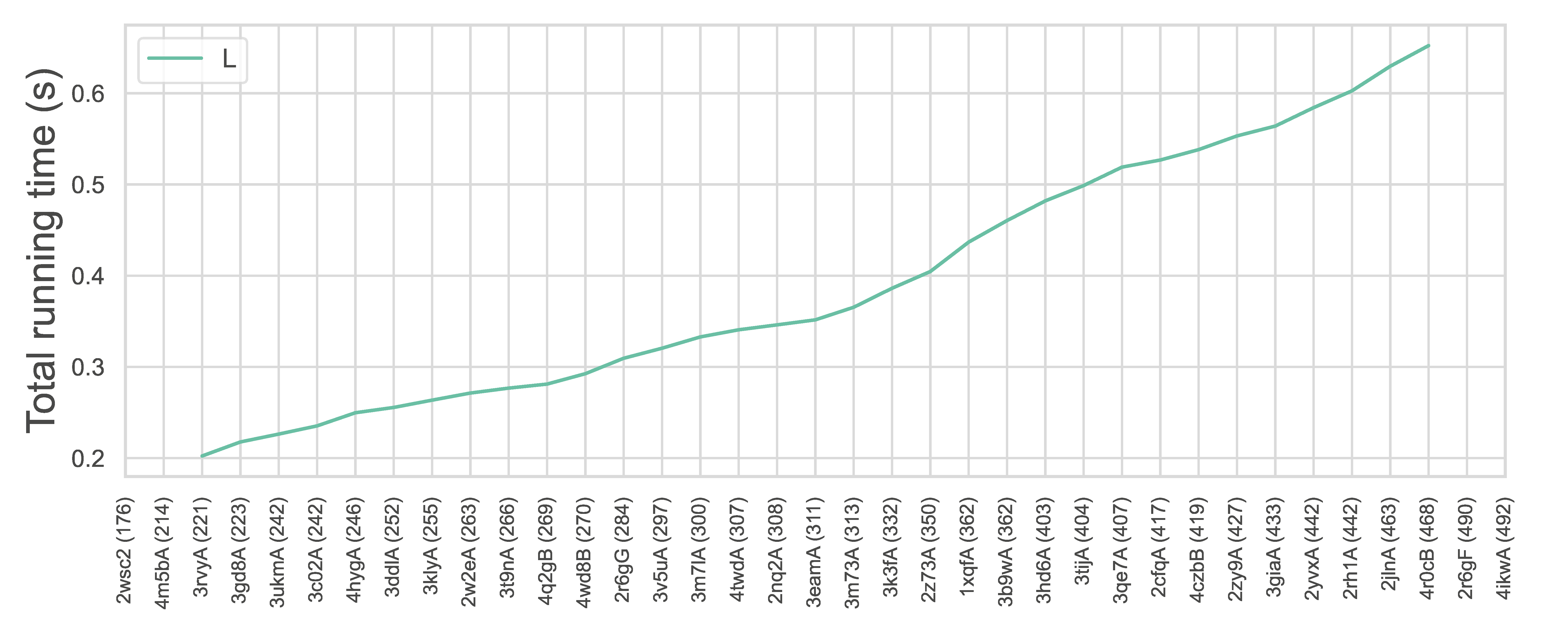


**Figure S3**. Total running time of generating cumuCCs per protein (PREVIOUS) at cut-off *L*.


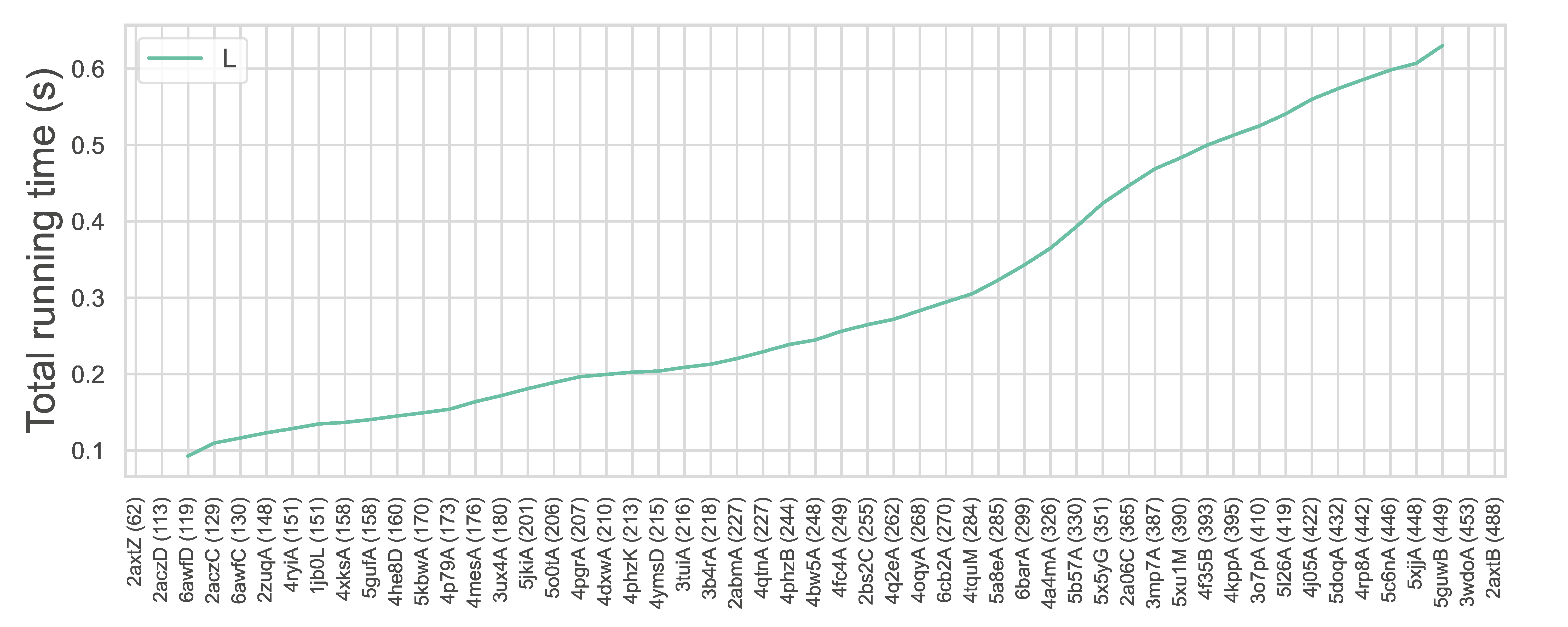


**Figure S4**. Total running time of generating cumuCCs per protein (TEST) at cut-off *L*.


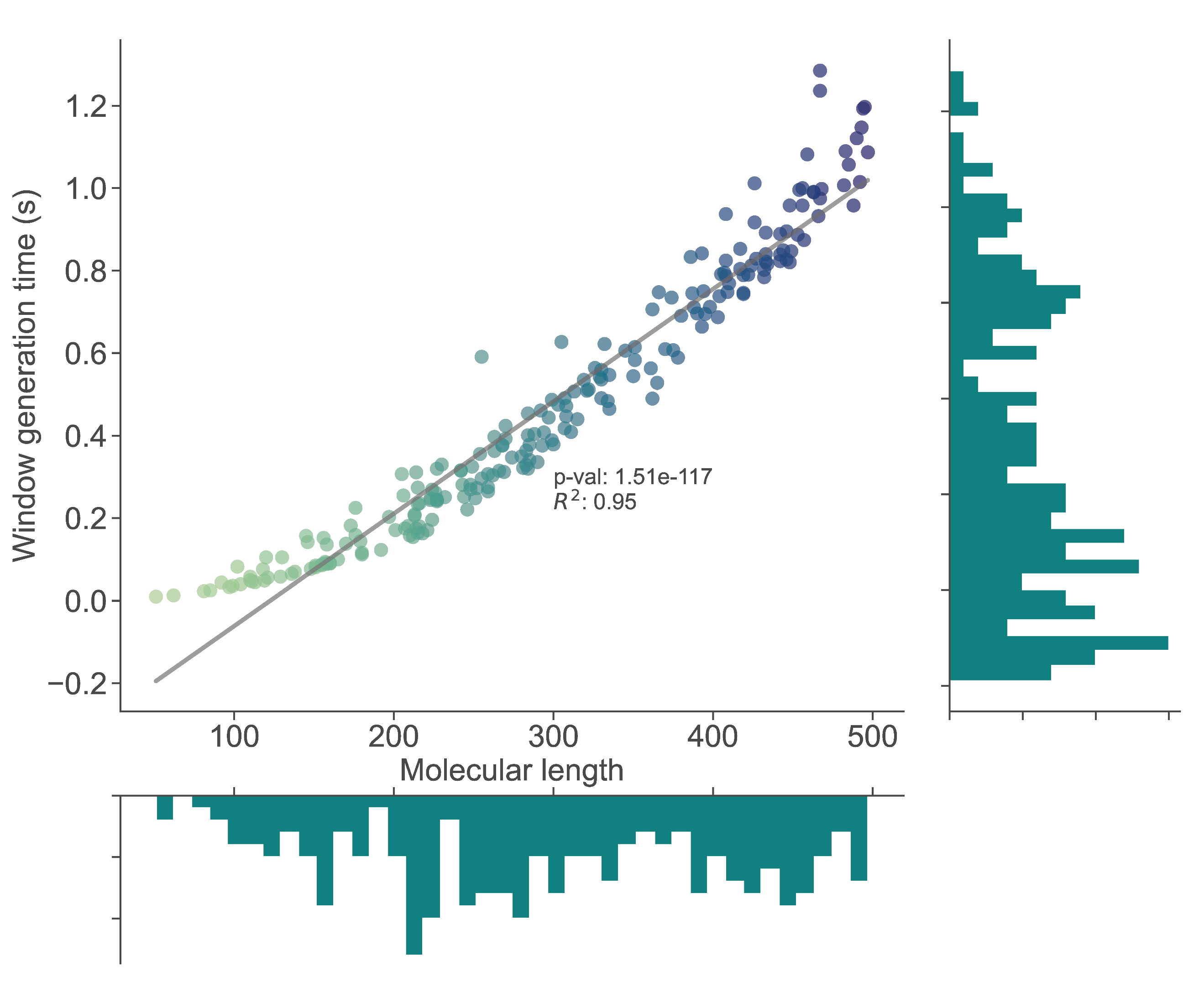


**Figure S5**. The running time for window generation *vs*. the molecular length per protein.


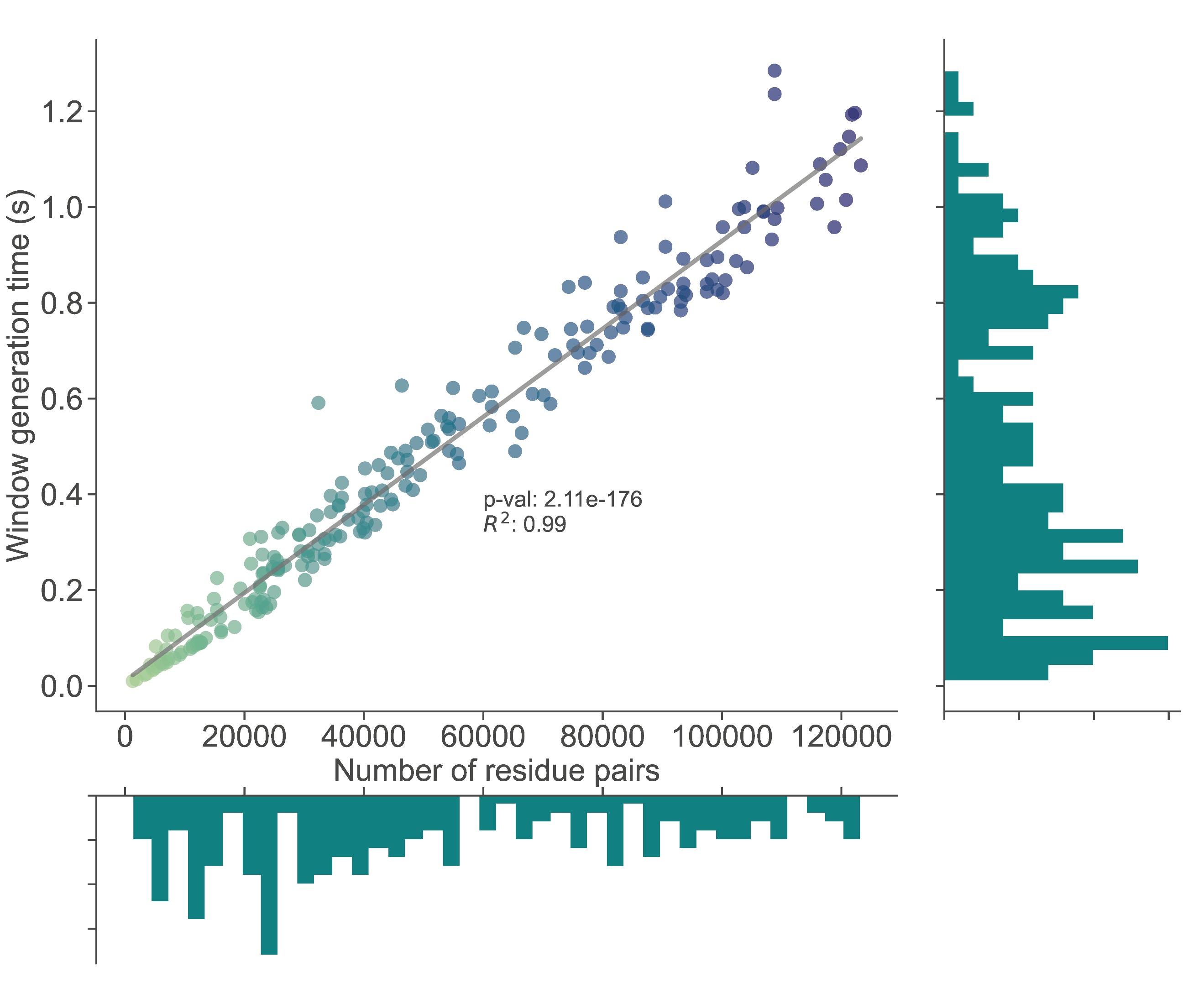


**Figure S6**. The running time for window generation *vs*. the number of residue pairs per protein.
